# Another Report of Acalculous Cholecystitis in a Greek Patient with Infectious Mononucleosis: A Matter of Luck or Genetic Predisposition?

**DOI:** 10.1155/2016/6080832

**Published:** 2016-01-14

**Authors:** Theocharis Koufakis, Ioannis Gabranis

**Affiliations:** Department of Internal Medicine, General Hospital of Larissa, 1 Tsakalof Street, 41221 Larissa, Greece

## Abstract

We here report a case of a young, male patient who presented with jaundice and was diagnosed with acalculous cholecystitis during the course of a primary Epstein-Barr Virus (EBV) infection. The coexistence of cholestatic hepatitis and acalculous cholecystitis in patients with infectious mononucleosis is extremely uncommon and only few cases can be found in the literature. Moreover, almost one-fourth of the total reports of this rare entity are coming from Greece. Whether this is a result of physicians' high index of suspicion due to previous reports or a consequence of genetic predisposition is an issue that deserves further investigation in the future. More studies are required in order to clarify the pathophysiological and genetic backgrounds that connect acalculous cholecystitis and EBV infection.

## 1. Introduction

Infectious mononucleosis (IM), first described by Sprunt and Evans in 1920, is a clinical entity caused by the Epstein-Barr Virus (EBV). EBV, also known as human herpesvirus 4, is a member of the herpes virus family, composed of a double helix of DNA of approximately 172,000 base pairs and containing about 85 genes [1]. Apart from IM, the virus has been associated with the pathogenesis of various diseases, including Burkitt's lymphoma, Hodgkin's lymphoma, gastric cancer, nasopharyngeal carcinoma, multiple sclerosis, and lymphomatoid granulomatosis [2].

IM is characterized by global geographical distribution, being more frequent among children and young adults [3]. Still, it can be diagnosed in patients of any age, from neonates to the elderly. The virus is transmitted through direct contact with an infected individual. In many cases, the infected subjects remain asymptomatic. When present, the characteristic clinical features include fever, pharyngitis, and lymphadenopathy [4]. Other common manifestations of the disease are fatigue, anorexia, nausea, splenomegaly, arthralgias, and myalgias.

Liver involvement is not unusual during the course of EBV infection, usually being mild and self-limited [5]. However, jaundice is an atypical presentation of the disease, observed only in 5% of patients with IM [6]. The exact pathophysiological background of jaundice development in IM is still uncertain. Cholestasis and virus-induced hemolysis have been proposed as the most probable mechanisms [5, 7]. Moreover, the coexistence of cholestatic hepatitis and acalculous cholecystitis in patients with IM is extremely uncommon and only few reports can be found in the literature.

## 2. Case Presentation

A 21-year-old male patient presented to the emergency department complaining about icteric skin 24 hours earlier. His past medical history was unremarkable and he was not on any medication. He denied any history of smoking or alcohol intake and there was no significant family history. Patient's physical examination revealed icteric skin and sclera. He did not mention unprovoked abdominal pain. Tenderness of upper right quadrant was noticed only during abdomen palpation (positive Murphy sign). Interestingly, no peripheral lymphadenopathy or exudative pharyngitis was observed. Low-grade fever was noticed during the time of hospitalization.

**Figure 1:**
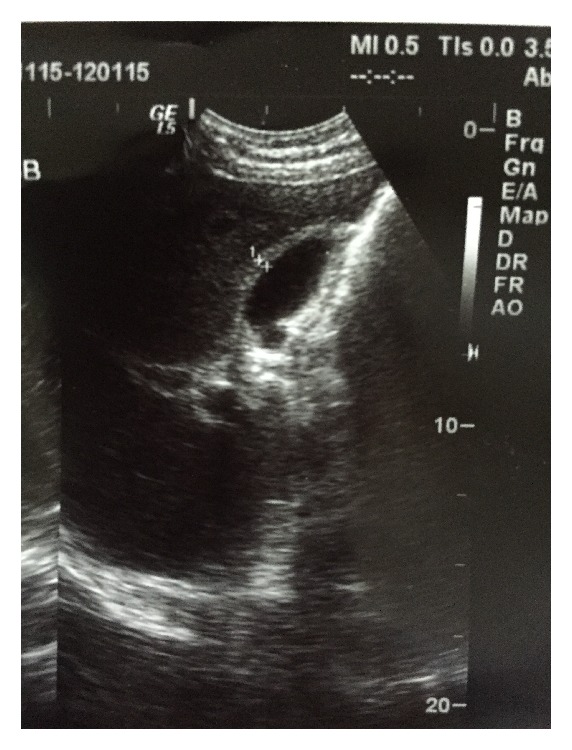
Abdominal ultrasound revealing thickening of the gallbladder wall and absence of stones or dilatation of the biliary tract.

Laboratory tests on admission were as follows: aspartate aminotransferase 172 IU/L (normal < 40 IU/L), alanine aminotransferase 232 IU/L (normal < 40 IU/L), total bilirubin 6.31 mg/dL (normal < 1 mg/dL), direct bilirubin 4.96 mg/dL (normal < 0.3 mg/dL), alkaline phosphatase 179 IU/L (normal < 140 IU/L), gamma-glutamyl transferase 350 IU/L (normal < 30 IU/L), lactate dehydrogenase 1141 IU/L (normal < 350 IU/L), prothrombin time 14.2 s (normal 11–14 s), international normalized ratio (INR) 1.07, C-Reactive Protein 3.3 mg/dL (negative < 0.5 mg/dL), and white cell count 11 × 10^3^ with 57% lymphocytes. Abdominal ultrasound revealed mild splenomegaly, thickening of the gallbladder wall (4.5 mm) (Figure 1), and positive sonographic Murphy sign. No stones, dilatation of the biliary tract, gallbladder swelling, or debris was detected.

The diagnosis of IM was established by high levels of IgM antibodies against EBV viral capsid antigen (>320 U/mL, normal < 20 U/mL). Viral hepatitis markers as well as antibodies for other hepatotropic viruses, human immunodeficiency virus (HIV) test, and blood and urine cultures proved to be negative.

No special treatment was administered apart from intravenous fluids. Our patient had an uneventful recovery and was discharged 10 days after admission, presenting significant improvement of his laboratory tests. Surgical intervention was thought to be unnecessary. In his follow-up visits, he remained in good physical condition and his liver tests gradually normalized within the next 2 months. Abdominal ultrasound repeated 30 days after admission demonstrated normal findings.

## 3. Discussion

Acute acalculous cholecystitis (AAC) is identified as the cause of about 5 to 10% of all cases of acute cholecystitis [12]. Being more frequent among patients with severe underlying conditions such as trauma and sepsis, it has been linked with higher percentages of mortality and complications when compared to the calculous type of the disease [13]. Though viral AAC is generally uncommon, several case reports of hepatitis A virus-induced AAC have been published in the literature [14, 15]. Direct invasion of the gallbladder wall by the EBV could be a possible explanation of the development of AAC during the course of IM [16]; still other mechanisms might be also involved. Attilakos et al. [9] discuss a potential contributing role of Gilbert's syndrome to the development of AAC in children with acute EBV infection. Fretzayas et al. [11] consider that biliary dyskinesia could be an alternative explanation of impaired gallbladder contractility in EBV infection.

Interestingly, the vast majority of patients previously reported with coexistence of AAC and EBV infection were females [16], suggesting a probable relationship between estrogens and the development of the disease [17]. Additionally, abdominal pain was prominent in most of the cases and it was the main reason that urged patients to seek medical advice. Differently, peripheral lymphadenopathy was generally absent, in contrast with the typical pattern of IM.

Most cases come from Europe and it is worth pointing out that 6 of them have been already reported from Greece [7–11] (Table 1). Whether this is a result of physicians' high index of suspicion due to previous reports or a consequence of genetic predisposition is an issue that deserves further investigation in the future.

It is worth mentioning that most of formerly described cases received antibiotics, while only one was subjected to cholecystectomy, because of severe clinical condition [18]. However, existing evidence suggests that supportive care and close monitoring of liver function tests are usually adequate management strategies for patients with EBV-induced AAC. The possibility of rash development associated with antibiotic treatment in subjects with IM [19] should also be considered.

Gallbladder wall thickening has been also reported in patients with acute hepatitis [20]. However, we consider that the wall thickening of patient's gallbladder in the reported case was a consequence of the developed AAC. Currently, there are no universally accepted criteria for the diagnosis of AAC. Gallbladder wall thickening of over 3 mm, distention of the gallbladder, localized tenderness, and pericholecystic fluid and sludge have been proposed as suitable ones [21, 22]. The combination of two or more of the above criteria, in a compatible clinical setting, is considered to be diagnostic [21]. Our patient met two of the reported criteria, that is, wall thickening (4.5 mm) and localized tenderness. Generally, a gallbladder wall thickness of at least 3.5 mm is accepted to be diagnostic of AAC [12].

In contrast with previous reports, our patient was a male and older than the majority of other cases. Moreover, he did not present with abdominal pain, but his main clinical manifestation was jaundice. Consistent with other cases, he did not develop pharyngitis and lymphadenopathy.

In conclusion, the coexistence of cholestatic hepatitis and acalculous cholecystitis represents a rare and atypical presentation of infectious mononucleosis. Physicians should be aware of this uncommon combination and suspect in time a possible EBV infection, in order to avoid unnecessary use of antibiotics or surgical procedures. Further studies are required in order to clarify the pathophysiological and genetic backgrounds that connect acalculous cholecystitis and EBV infection.

## Figures and Tables

**Table 1:** Main characteristics of cases with EBV-induced acute acalculous cholecystitis (AAC), reported from Greece.

1st author, year (ref.)	Age, sex	ALT (IU/L)	Total bilirubin	ALP (IU/L)	Surgical intervention
Prassouli, 2007 [7]	13, female	674 (5–45)	4 mg/dL	721 (<248)	No
Lagona, 2007 [8]	4, female	304 (5–45)	4.6 mg/dL	236 (38–148)	No
Attilakos, 2009 [9]	5, male	257 (5–45)	1.8 mg/dL	919 (38–148)	No
Cholongitas, 2009 [10]	19, female	584	6.5 mg/dL	710	No
Fretzayas, 2014 [11]	11, female	198	31 *μ*mol/L	536	No
Fretzayas, 2014 [11]	12, female	195	Not provided	Not provided	No
Present case	21, male	232 (<40)	6.31 mg/dL	179 (<140)	No
